# Impact of Climate Change on Emerging Infectious Diseases and Human Physical and Mental Health in Bangladesh

**DOI:** 10.1002/hcs2.129

**Published:** 2025-02-13

**Authors:** Md. Siddikur Rahman, Afiya Antara Anika, Rafia Amin Raka, Ajlina Karamehic Muratovic

**Affiliations:** ^1^ Department of Statistics Begum Rokeya University Rangpur Bangladesh; ^2^ Department of Sociology and Anthropology St Louis University St Louis Missouri USA

**Keywords:** Bangladesh, mental health, respiratory illnesses, waterborne diseases

## Abstract

This study aims to give possible solutions to the impact of climate change on the nation's physical and mental health and emerging infectious diseases. Improving Bangladesh's healthcare, response, and data collection systems is a public health emergency.

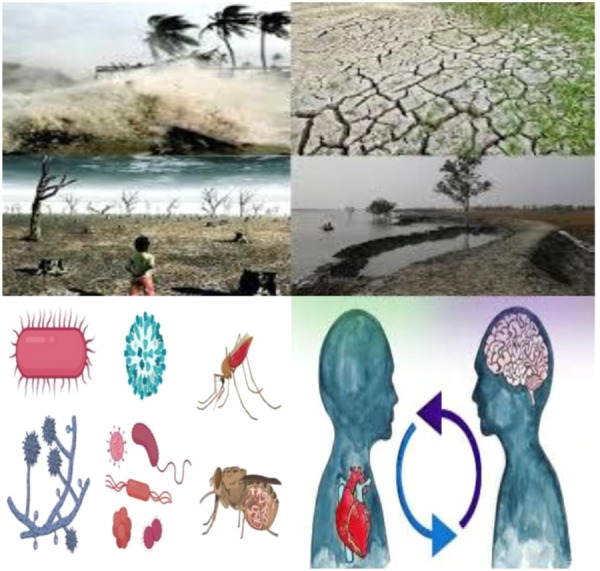

**Abbreviations**
ANN: artificial neural network
COVID‐19: coronavirus disease
IoT: Internet of Things
ML: machine learning
PTSD: posttraumatic stress disorder

## 1 Introduction

**Figure 1:**
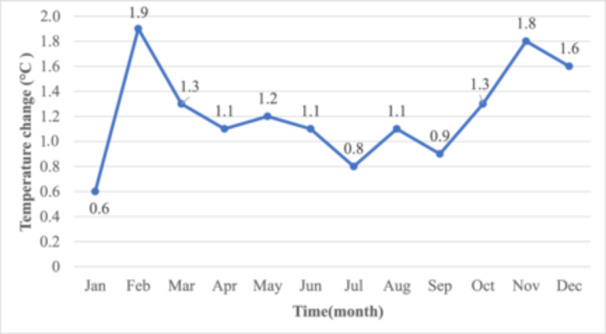
Change in mean historical monthly temperature (°C) between 1901 and 2019. *Data Source:* Climate Change Knowledge Portal [3]. *Note:* The mean temperature difference is based on two 30‐year averages: 1901–1930 and 1991–2019.

Climate change is one of the most significant and widespread global issues, contributing to emerging infectious diseases and threatening human physical and mental health [1, 2]. Bangladesh, one of the most densely populated countries in South Asia, experiences unpredictable weather and a steady increase in temperature and precipitation. Between 1901 and 2019, Bangladesh saw an average temperature increase of 0.5°C according to the change in mean monthly temperatures. Warming is more pronounced in winter months, such as January (from 0.6°C to 1.9°C) and November (1.3°C to 1.8°C), compared to smaller increase during the monsoon (Figure 1). This reflects uneven seasonal warming, driven by climate change, with winter and pre‐winter months experiencing the most significant temperature rises.

Climate change jeopardizes both human physical and mental health of Bangladesh's residents [4], increasing the prevalence and variety of infectious diseases as well as mental health problems including depression and anxiety [5, 6, 7]. Evidence from Bangladesh suggests that climate change is expected to have even greater impact on population mental health in the coming decades, and climate actions need to be taken now [8, 9, 10].

The following sections explore the relationship between climate change and emerging infectious diseases, while also examining deeper the ways climate change affects human and physical health. Conclusions regarding how the government can institute appropriate policies to tackle the issue of climate change are offered.

## 2 Climate Change and Emerging Infectious Diseases

Climate change is documented to have worsened over half of all known emerging infectious diseases that threaten humans [11]. A wealth of literature discusses climate change's complexity and potential effects on emerging infectious diseases, including vector‐borne, water‐borne, air‐borne, and food‐borne diseases [12, 13]. Malaria, diarrheal disease, enteric fever, encephalitis, pneumonia, and bacterial meningitis are six common climate‐sensitive emerging infectious diseases in northeastern Bangladesh [14]. More recently, COVID‐19 has exacerbated human health in this nation and has been linked to the negative effects of climate change [15, 16]. Numerous bacteria cause gastrointestinal diseases and illnesses, including respiratory and pulmonary infections, influenza, malaria, tuberculosis (TB), dengue, and chikungunya, with high morbidity and mortality rates in Bangladesh [17]. There are also concerns about zoonotic illnesses in this country. For instance, the encephalitis caused by the Nipah virus has a fatality rate of 75%, avian influenza is a major problem for poultry, and some illnesses can coexist with acute anthrax epidemics in grazing animals like cattle [18]. On the other hand, rising humidity and heat will likely result in a decline in water‐borne illnesses like cholera. For instance, a 1°C increase in mean temperature reduces the probability of respiratory illnesses by 4.2%. In contrast, a 1% rise in relative humidity results in a 1.6% decrease in the risk of contracting water‐borne infections [19]. In 2019, high rainfall before the monsoon brought the largest dengue epidemic, with 101,354 cases reported from 2000 to 2018 [17]. Additionally, temperature, humidity, and rainfall all play a role in TB incidence, making tuberculosis a major public concern in the country [20].

In the 21st century, a greater proportion of deaths have been caused by emerging and re‐emerging infections than by seasonal and endemic infections [21, 22, 23]. Therefore, Bangladesh needs to take urgent action by strengthening health systems to avert and reduce outbreaks of climate‐sensitive diseases. Upgraded data collection and smart disease surveillance systems can help track and predict the evolution of potential disease outbreaks [19]. Smart disease surveillance equipment, illness diagnostics, and disease forecasting can all benefit greatly from digital healthcare provision and surveillance systems known as the Internet of Things (IoT) and machine learning (ML) [24]. To successfully mitigate these global health challenges, it is essential to create transdisciplinary strategies, including one‐health approaches that consider the social, economic, and environmental components of the problem.

## 3 Climate Change and Human Physical and Mental Health

Due to climate change, risks to human physical and mental health are increasing in Bangladesh [25, 26]. For example, skin diseases, headaches, heatstroke, cough, and fever are frequently occurring physical health problems due to rising temperatures and changing rainfall patterns. Contaminated groundwater causes malnutrition, scarcity of safe water, hypertension, and premature birth. Furthermore, stagnant weather conditions cause cardiovascular, respiratory, and allergy diseases [27].

Along with effects on physical health, substantial negative consequences on mental health have also been documented in the country. Emotional exhaustion, eco‐anxiety, eco‐depression, eco‐grief, posttraumatic stress disorder (PTSD), sleep difficulties, and suicidal ideation are climate change's most common effects on human mental health that have been observed [1, 19, 27, 28]. Additionally, Bangladesh has a higher suicide rate, positioning it as the second country with the most suicides linked to mental stress [28].

Floods are one of nature's main destructive disasters in the country, with significant economic and health effects [28, 29]. Trauma and shock are two of the most prevalent psychological reactions documented following a severe climate change disaster, such as tornadoes and floods [5]. It is well documented that trauma and shock can initiate a variety of emotional, cognitive, and physical responses that can contribute to mental stress and make one more vulnerable to PTSD, chronic mental health challenges, and even physical morbidity [28, 29, 30].

A tornado in the Tangail district in 1996 affected 66% of the population, leading to long‐lasting trauma and an increased demand for psychological treatment. Previous studies have found that among cyclone Sidr survivors, 25% suffer from PTSD, 18% from somatoform disorder, 16% from mixed anxiety, and 15% from major depressive disorders [19].

As part of the National Mental Health Strategy 2020–2030, the Government of Bangladesh has committed to offering top‐notch mental health services and information in a cross‐sectoral, integrated, and responsive approach. However, despite developing legal documents on adaptation, Bangladesh lacks the institutional structure and capacity to incorporate climate change adaptation [27]. Collaboration among government, businesses, nonprofits, local policymakers, news media, and other professionals is vital to advancing climate resilience action and its implementation [5]. Additionally, building upon global commitments and closing the large funding gap that exists for mental health and psychosocial support can help minimize the detrimental effects of climate change on mental health [25].

## 4 Conclusion

This article examined how climate change affects infectious diseases in Bangladesh, subsequently influencing the physical and mental health of its population. The connection between infectious diseases and mental health is complex and a discussion beyond this study; however, it has been well‐established that infections can trigger various psychiatric symptoms [31]. The paper ultimately urges different government departments to adopt appropriate policies to tackle the issue of climate change and suggests a multidisciplinary approach to address physical and mental health challenges related to climate change. The paper highlights a large gap in an urgent issue of climate‐sensitive diseases and mental health‐related diseases in Bangladesh. Climate change severely affects Bangladesh because of a lack of institutional, social, technological, and economic resources. A climate change resilient healthcare system, including proper policy and its practical implementation, must be developed through strong strategic planning that includes all stakeholders, adequate funding, and multi‐sectoral cooperation. To combat pathogen emergence and dissemination, reducing disparities in access to healthcare and enhancing disease surveillance for communicable diseases should be prioritized. Integrated support from the commercial sector and international organizations is vital. Addressing educational programs regarding climate change for general people and healthcare providers is also critical.

## Author Contributions

**Md. Siddikur Rahman:** conceptualization (equal), data curation (equal), formal analysis (equal), funding acquisition (equal), investigation (equal), methodology (equal), project administration (equal), resources (equal), software (equal), supervision (equal), validation (equal), visualization (equal), writing–original draft (equal), writing–review and editing (equal). **Afiya Antara Anika:** data curation (equal), visualization (equal). **Rafia Amin Raka:** data curation (equal), resources (equal), visualization (equal). **Ajlina Karamehic Muratovic:** investigation (equal), project administration (equal), supervision (equal).

## Ethics statement

The authors have nothing to report.

## Consent

The authors have nothing to report.

## Conflicts of Interest

The authors declare no conflicts of interest.

## Data Availability

The datasets used and analysed during the current study are available from the corresponding author upon reasonable request.
